# Chemical Characterisation and Antihypertensive Effects of Locular Gel and Serum of *Lycopersicum esculentum* L. var. “Camone” Tomato in Spontaneously Hypertensive Rats

**DOI:** 10.3390/molecules25163758

**Published:** 2020-08-18

**Authors:** Paola Marcolongo, Alessandra Gamberucci, Gabriella Tamasi, Alessio Pardini, Claudia Bonechi, Claudio Rossi, Roberta Giunti, Virginia Barone, Annalisa Borghini, Paolo Fiorenzani, Maria Frosini, Massimo Valoti, Federica Pessina

**Affiliations:** 1 Department of Molecular and Developmental Medicine, University of Siena, Via Aldo Moro 2, 53100 Siena, Italy; paola.marcolongo@unisi.it (P.M.); alessandra.gamberucci@unisi.it (A.G.); roberta.giunti@unisi.it (R.G.); virginia.barone@unisi.it (V.B.); a.borghini12@gmail.com (A.B.); 2 Department of Biotechnology, Chemistry and Pharmacy, University of Siena, Via Aldo Moro 2, 53100 Siena, Italy; gabriella.tamasi@unisi.it (G.T.); pardini4@student.unisi.it (A.P.); claudia.bonechi@unisi.it (C.B.); claudio.rossi@unisi.it (C.R.); 3 Department of Medicine, Surgery and Neurosciences, University of Siena, Viale Bracci 16, 53100 Siena, Italy; paolo.fiorenzani@unisi.it; 4 Department of Life Sciences, University of Siena, Via Aldo Moro 2, 53100 Siena, Italy; maria.frosini@unisi.it (M.F.); massimo.valoti@unisi.it (M.V.)

**Keywords:** rats, inbred SHR, blood pressure, antihypertensive agents, antioxidants, tomatine, polyphenols

## Abstract

Blood pressure control in hypertensive subjects calls for changes in lifestyle, especially diet. Tomato is widely consumed and rich in healthy components (i.e., carotenoids, vitamins and polyphenols). The aim of this study was to evaluate the chemical composition and antihypertensive effects of locular gel reconstituted in serum of green tomatoes of “Camone” variety. Tomato serum and locular gel were chemically characterised. The antihypertensive effects of the locular gel in serum, pure tomatine, and captopril, administered by oral gavage, were investigated for 4 weeks in male spontaneously hypertensive and normotensive rats. Systolic blood pressure and heart rate were monitored using the tail cuff method. Body and heart weight, serum glucose, triglycerides and inflammatory cytokines, aorta thickness and liver metabolising activity were also assessed. Locular gel and serum showed good tomatine and polyphenols content. Significant reductions in blood pressure and heart rate, as well as in inflammatory blood cytokines and aorta thickness, were observed in spontaneously hypertensive rats treated both with locular gel in serum and captopril. No significant effects were observed in normotensive rats. Green tomatoes locular gel and serum, usually discarded during tomato industrial processing, are rich in bioactive compounds (i.e., chlorogenic acid, caffeic acid and rutin, as well as the glycoalkaloids, α-tomatine and dehydrotomatine) that can lower in vivo blood pressure towards healthier values, as observed in spontaneously hypertensive rats.

## 1. Introduction

High blood pressure is a major risk factor for cardiovascular disease, including myocardial infarction and stroke [1]; visceral obesity, plus any two among hypertension, high fasting blood glucose levels, or dyslipidemia are risk factors for type 2 diabetes and metabolic syndrome.

The latter is currently a primary health problem worldwide [2], affecting 20–25% of the adult population according to the International Diabetes Federation consensus worldwide definition of metabolic syndrome.

Chronic treatments with commonly used antihypertensive drugs reduce the risk of total major cardiovascular events, but have high medication costs and risk of adverse reactions, which may be severe. A recent study [3] found an association between the pharmacological treatment of patients with mild hypertension and an increased risk of adverse events, including hypotension, syncope, electrolyte abnormalities and acute kidney damage. An alternative or supplementary treatment that combines efficacy with low therapeutic costs, especially for people with borderline to mild hypertension, is a dietary approach to controlling blood pressure and decreasing the risk of hypertension-related complications [4]. Dietary management can be combined or even made more effective by adding specific nutrients to traditional therapy to achieve additive or even synergistic effects.

The advantages of a natural diet can be ascribed to a combination of bioactive compounds [5] or to specific “functional foods”. A growing number of scientific papers is continuously being published on this topic [6,7,8,9], together with papers relevant to nutraceutical molecules and delivery systems able to circumvent their common low bioavailability [10,11].

The tomato (*Solanum lycopersicum* L.; *Lycopersicum esculentum* L.) is the fruit of an herbaceous plant of the Solanaceae family. This widely consumed vegetable is high in antioxidants and other bioactive components, such as vitamins E and C, flavonoids (e.g., rutin), phenolic compounds (e.g., chlorogenic acid), carotenoids (β-carotene and lycopene) and glycoalkaloids (tomatine) [12,13,14,15]. Moreover, tomatoes are the main source of lycopene in the human diet. All these tomato components have demonstrated strong protective effects against cancer [16] and cardiovascular disease [17], ischemia/reperfusion damage [18], as well as strong antioxidant properties [19]. Besides the well-known healthy properties of red (mature) tomatoes, immature green tomatoes contain low levels of carotenoids and high levels of a different family of secondary metabolites, known as steroid glycoalkaloids, such as α-tomatine and dehydrotomatine [20]. Commonly found in a ratio of 10:1, respectively, together they are known as tomatine. The biological activity of tomatine has been widely reported, particularly its lipid lowering, anti-carcinogenic and anti-inflammatory effects [21].

Locular gel is the portion of tomato fruits consisting of the jelly-like material surrounding the seeds, dispersed in aqueous medium (henceforth serum). In the food industry, when producing tomato concentrate, pulp and sauces, the locular gel is often a by-product (a waste) discarded at the seeds-removing stage. The locular gel was manually separated from the fruits and gently filtered to be separated by the seeds, and finally centrifuged to be separated from the serum and possible pulp traces. A previous study reported that the locular gel of tomatoes of the Camone variety is the part of the fruit (as distinct from skin and pulp) richest in the two glycoalkaloids α-tomatine and dehydrotomatine and high in polyphenols, of which chlorogenic acid is the most abundant [15]. Locular gel could therefore be a product of nutraceutical interest, useful for fortifying foods or as a dietary supplement.

The aim of present study was: (1) the chemical characterisation of the tomato locular gel and of the serum obtained from green tomatoes of the Camone variety; (2) the evaluation of the antihypertensive effects of the combined gel-serum (Gs) on spontaneous hypertensive rats (SHRs) and normotensive Wistar Kyoto rats (WKYs) used as age-matched control strain. The effects of daily administration of Gs was compared with the effects of pure tomatine (T) and the angiotensin-converting enzyme (ACE) inhibitor captopril (C) (positive control). Plasma glucose, plasma triglycerides, blood inflammatory cytokines, aorta thickness and liver metabolising activity were assessed.

## 2. Results

### 2.1. Chemical Characterisation of Camone Tomato Locular Gel and Serum

**Figure 1:**
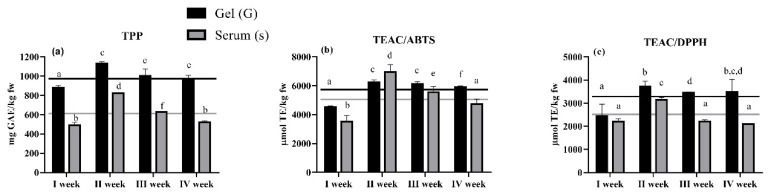
Total polyphenol content and antioxidant activity of gel (G) and serum (s) from Camone tomatoes sampled in four different weeks. (**a**) TPP (mean ± SD mg GAE/kg fw, *n* = 27); (**b**) and (**c**) AOA (mean ± SD μmol TE/kg fw, *n* = 27) measured by TEAC/ABTS and TEAC/DPPH assays, respectively. Bars with different letters in each histogram, are significantly different (ANOVA with Tukey post-test, *p* < 0.05). Horizontal lines represent mean values in the four weeks of sampling. TPP: Total polyphenol content; AOA: antioxidant activity; TEAC: trolox equivalent antioxidant capacity.

Total polyphenol content (TPP) and antioxidant activity (AOA) of gel and serum from Camone tomatoes in four weeks of sampling are shown in Figure 1.

The TPP in gel averaged 993.5 ± 105.5 mg GAE/kg fw. The average AOA determined by TEAC/ABTS or TEAC/DPPH assays was 5704 ± 797.5 and 3270 ± 573.1 µmol TE/kg fw, respectively. TPP and AOA of serum showed slightly lower average values, but with the same trend as observed for gel (average TPP 614.7 ± 149.7 mg GAE/kg fw; average TEAC/ABTS 5182 ± 1438 µmoL TE/kg fw; average TEAC/DPPH 2413 ± 494.2 µmoL TE/kg fw). The two measures were rather uniform over the four weeks of sampling, except for the gel and serum in week 2, which showed slightly higher values. Selected polyphenols and glycoalkaloids were quantified by HPLC-ESI-QqQ-MS/MS analysis (Table 1).

Chlorogenic acid was the most abundant polyphenol in all samples, ranging from 29.5 ± 0.6 (Week 4) to 90.7 ± 3.5 mg/kg fw (Week 2) in gel and from 26.1 ± 0.8 (Week 4) to 92.3 ± 4.7 mg/kg fw (Week 2) in serum. These concentrations were about one order of magnitude higher than those of caffeic acid, a biosynthetic precursor of chlorogenic acid. Lower concentrations of *p*-coumaric acid and rutin and traces of kaempferol-3-*O*-rutinoside were also detected. It is interesting that the gel and serum from the same week of sampling showed similar concentrations of polyphenols. The variation in the phenol composition of samples collected in different weeks may be due to the natural biological variability of the fruits, despite the fact that they were cultivated in the same area with the same agricultural practices and harvested at the same stage of ripening.

Regarding glycoalkaloids content, there was a significant decrease in the content of α-tomatine (*p* < 0.05) in gel samples in different weeks, passing from 61.7 ± 0.9 mg/kg fw (Week 1) to 4.41 ± 0.32 mg/kg fw (Week 4). Dehydrotomatine content was about one tenth that of α-tomatine in all samples. These decreases can be explained by the fact that even small variations in ripening stage may lead to a reduction in the biosynthesis of the two glycoalkaloids.

The concentration of the major analytes in Gs samples, calculated as the sum of the results obtained for gel and serum expressed as the μg/kg body weight of rats and used for rat gavaging, showed a decreasing trend over the four weeks of treatment (Table 2).

### 2.2. Animal Experiments: Effects of Four-Week Treatment with Vehicle (V), Gel/Serum (Gs), Tomatine (T) and Captopril (C)

#### 2.2.1. Physiological Parameters

The body weight of SHRs was slightly lower than that of control WKYs, as expected from the growth chart of the supplier. However, none of the treatments had any effect on the growth rate of either group (Table 3).

Heart weights are reported in Table 3. SHRs V heart weight was significantly higher than that of matched WKYs (*p* < 0.05, Student t-test). Notably, all the treatments decreased heart weight in SHRs being not significantly different from matched WKYs.

None of the four-week treatments caused any deaths in either type of rat. No signs of changes in the skin, fur, eyes mucous membranes or salivation were observed, suggesting that the treatments and doses are safe.

Blood glucose and triglyceride levels measured in blood serum were not significantly different in SHRs and WKYs, and none of the treatments affected these parameters (Table 4). Similarly, measured urine parameters (data not shown) and urine volumes (Table 4) did not differ between the experimental groups.

#### 2.2.2. Systolic Blood Pressure (SBP) and Heart Rate (HR)

**Figure 2:**
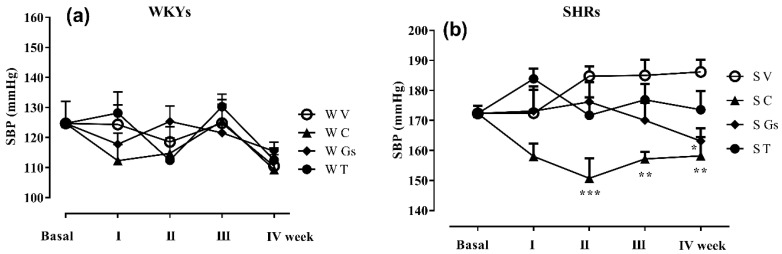
Effect of different treatments on systolic blood pressure (SBP) in SHRs and WKYs. Animals were treated for 4 weeks with vehicle, captopril (50 mg/kg), tomatine (8 mg/kg) or gel/serum (12.40 g/kg). Panel (**a**) and (**b**): time course of SBP in WKYs and SHRs, respectively, assessed at day 0 (before the treatment, basal) and at the end of week 1, 2, 3 and 4 of treatment. * *p* < 0.05, ** *p* < 0.01, *** *p* < 0.001 vs. SV at the same time (two-way ANOVA followed by Bonferroni post-test). V: vehicle; C: captopril; T: tomatine; Gs: gel/serum. SHRs: S; WKYs: W.

C and Gs treatments significantly decreased SBP in SHRs, while no effect was detected for T at the end of the treatment period, even if the value was lower than in SHRs V (Figure 2). None of the treatments exerted any significant effects in WKYs. It is interesting that while C lowered SBP after only one week of treatment, Gs began to affect SBP in Week 3, showing a highly significant effect at the end of Week 4 (Figure 2b).

**Figure 3:**
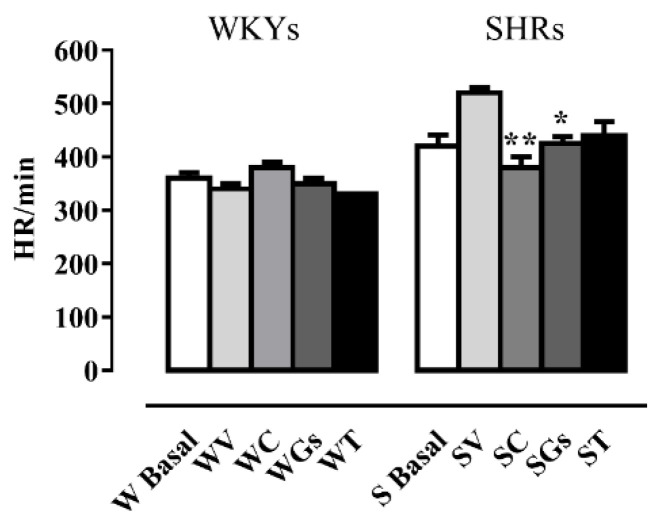
Effect of different treatments on heart rate (HR) in SHRs and WKYs. Rats were treated for 4 weeks with vehicle, captopril (50 mg/kg), tomatine (8 mg/kg) and gel/serum (12.40 g/kg). Data reported as mean ± SEM, was measured at the beginning (basal) and at the end of four weeks of treatment. * *p* < 0.05; ** *p* < 0.01 vs. SV (one-way ANOVA followed by Bonferroni post-test). V: vehicle; C: captopril; T: tomatine; Gs: gel/serum. SHRs: S; WKYs: W.

Heart rate was significantly higher in SHRs than in WKYs (520 ± 14 vs. 340 ± 13, respectively, Student t-test *p* < 0.001) at the end of the four weeks of treatment with vehicle. Captopril and Gs treatment also significantly decreased HR in SHRs. None of the treatments exerted any statistically significant effect on WKYs (Figure 3).

#### 2.2.3. Histological Analysis

**Figure 4:**
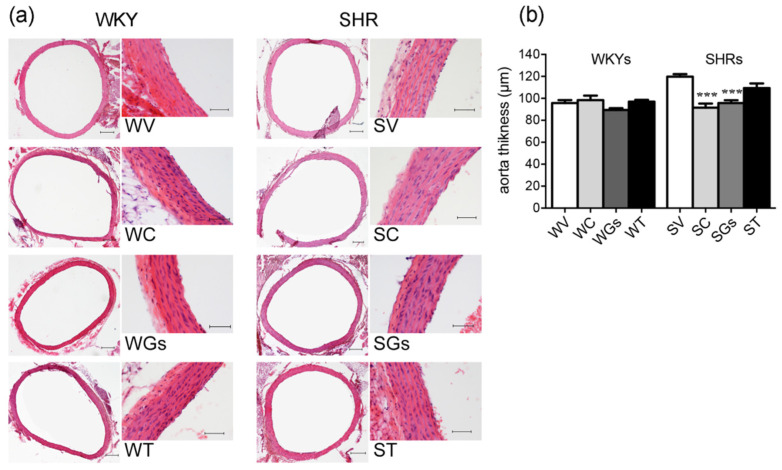
Histological evaluation of aorta wall thickness. Aorta samples obtained after administration vehicle, captopril (50 mg/kg), tomatine (8 mg/kg) and gel/serum (12.40 g/kg) from WKYs and SHRs. (**a**) Aorta slices were stained with hematoxylin and eosin and observed under a microscope. Scale bar at low magnification = 200µm, scale bar at high magnification = 50 µm. (**b**) Bar graph reports aorta thicknesses as mean ± SEM. *** *p* < 0.001 vs. SV (one-way ANOVA followed by Bonferroni post-test). V: vehicle; C: captopril; T: tomatine; Gs: gel/serum. SHRs: S; WKYs: W.

Aorta wall thickness significantly increased in SHRs V as compared to WKYs V (Student t-test; *p* < 0.001). While treatment with C, Gs and T had no effect in the WKY group, in SHRs C and Gs treatments were associated with a decrease in aorta thickness. No significant difference was observed with T treatment (Figure 4).

#### 2.2.4. Serum Inflammatory Cytokine

**Figure 5:**
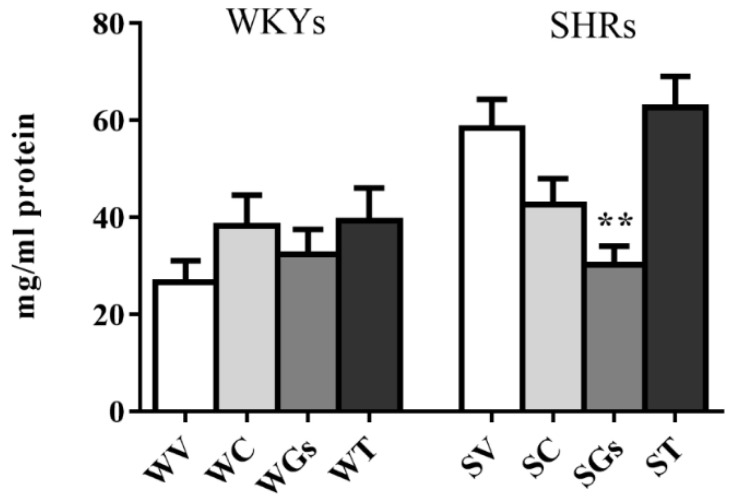
Effect of different treatments on TNF-α plasma content in SHRs and WKYs. Animals were treated for 4 weeks with vehicle, captopril (50 mg/kg), tomatine (8 mg/kg) and gel/serum (Gs) (12.40 g/kg). Data are reported as mean ± SEM. ** *p* < 0.01 vs. SV (one-way ANOVA followed by Bonferroni post-test). V: vehicle; C: captopril; T: tomatine; Gs: gel/serum. SHRs: S; WKYs: W.

The proinflammatory cytokine TNFα was significantly elevated in SHRs as compared to WKYs and was significantly reduced by treatment with Gs, reaching values similar to those recorded in WKYs treated with vehicle and Gs (Figure 5).

Contrastingly, no effect was observed with captopril and tomatine treatment. Other cytokines, such as IL-6 and IL-1β, were not detectable (data not shown).

#### 2.2.5. Liver Cytochrome P450- and b5-content, NADPH-Cytochrome P450 Reductase Activity

As reported in Table 5 and Table 6, none of the treatments used in this study affected microsomal CYP content and related activities. Interestingly, SHRs showed almost double the reductase activity of WKYs, although the difference was not significant. Reductase activity reflected benzyl-*O*-demethylase activity which was higher in all SHR groups than in WKYs (*p* < 0.05, Student t-test, WV vs. SV). No significant differences were observed between groups of the same strain, as assessed by ANOVA.

## 3. Discussion

Here we investigated the efficacy of Gs from Camone tomatoes, in the treatment of hypertension. Tomato properties have already been described and tomato extracts revealed ACE- and platelet-aggregation-inhibiting effects [22]. Tomato extracts are particularly rich in antioxidants (e.g., carotenoids such as lycopene and vitamin E) which help to reduce blood pressure in patients with mild hypertension never treated with antihypertensive drugs [18]. Hamsters on a tomato-supplemented diet for three weeks showed a significant reduction in LDL cholesterol and triglyceride levels [23]. It is well known that high LDL and triglyceride levels, high blood pressure, obesity and hyperglycemia are a multiplex risk factor for coronary heart disease and cardio-vascular complications [2].

In this study, our attention was mainly focused on the blood pressure lowering effects of tomatoes of the Camone variety. We investigated the Gs, which surrounds the seeds, normally discarded during industrial processing of tomato concentrate, tomato pulp and tomato sauces, and recently chemically characterised [15].

We used spontaneously hypertensive rats, a well-known animal model of hypertension. These rats are considered suitable for studying the antihypertensive properties of drugs or dietary supplements as the development of hypertension is very similar to that in humans [24].

The experimental design involved supplementing a standard chow diet with Gs for four weeks. The rats were treated starting at ten weeks of age, when systolic blood pressure is already slightly elevated, i.e., a condition of mild hypertension. Blood pressure in untreated SHRs increases with age. Rats were treated by oral gavage, so the amounts administered were accurate. Our research shows, for the first time, that a quite prolonged treatment with Gs induced a significant decrease in blood pressure, similar to that caused by the antihypertensive drug captopril, with respect to untreated SHRs. The effect of Gs was significant at the end of Week 4, while captopril lowered blood pressure already at Week 2. Tomatine showed a similar trend to Gs, but blood pressure reduction was not significantly different from that of SHRs treated with vehicle.

The long treatment necessary to show the effect of Gs depends on the fact that the used fraction is not a drug. This suggests that tomato Gs supplement can be used as continuous preventive treatment to prevent an increase in blood pressure. If the supplement is suspended, it is plausible that blood pressure slowly recover, as happens after suspending *Fraxinus excelsior* L. seed extract treatments [24].

This and our previous studies showed that these tomato fractions are rich in bioactive compounds and have high antioxidant capacity. Many studies, including meta-analyses [17], have dealt with the antihypertensive effects of lycopene and tomato extracts that are mainly ascribed to their strong antioxidant activity. In our case, Gs was obtained from the Camone tomato variety that differs in chemical components from red tomatoes, while maintaining a high antioxidant capacity, as indicated in Figure 1. Camone tomatoes are particularly rich in chlorogenic acid, an important biologically active dietary polyphenol constituent of green coffee [25], and in the glycoalkaloid tomatine. Chlorogenic acid and its hydrolysis product caffeic acid produced variable results in studies regarding blood lipids/cholesterol and glucose levels [25]. Chlorogenic acid has already been reported as potentially active in reducing blood pressure in SHRs [25], while tomatine could have effects in reducing cholesterol and triglyceride levels in plasma [23,26], but no data are available for its effects on blood pressure. Flavonoids, such as rutin, occur in low concentrations in Gs and are known to have ACE-inhibitory activities [27].

Tomatine is a chemical compound found in Gs at variable concentrations. Our chemical analysis showed that some compounds that are abundant in Gs of green tomatoes (tomatine and to a lesser extent chlorogenic acid) diminish in concentration as the fruit ripens, unlike what happens to other components of the pulp and the skin of red tomatoes, such as carotenoids, including lycopene.

It is plausible that the anti-hypertensive effect shown by Gs was due to more than one component or to an additive effect of polyphenols and glycoalkaloids, which need to be administered for a long period of time. The effects of Gs could be more pronounced if treatment is started before the onset of hypertension and continued at length, as indicated in a recent paper on the anti-hypertensive effect of an enriched olive oil, that produced a reduction in blood pressure after five weeks of treatment and reduced cardiac hypertrophy after eight weeks of treatment [4,28]. A similar observation was also reported by other authors [29].

These findings are evidence that Gs significantly decreases heart rate to an extent similar to captopril in SHRs, as shown in Figure 2. This is very important, since a decrease in blood pressure and heart rate are associated with vascular and heart protection [29]. Indeed, Gs may also help maintain the normal vascular anatomy and structure of arterial blood vessels. Interestingly, aorta thickness was significantly greater in SHRs than in WKYs both treated with vehicle. This was not evident in SHRs treated with Gs, whose aortas were similar to those of rats treated with captopril (Figure 4). Captopril also counteracted SHR heart weight gain; similarly, SHRs treated with Gs had a lower heart weight than SHRs treated with V, but the difference was not significant. Four weeks of Gs treatment may not be enough to restore all cardiovascular parameters, especially those linked to morphological modifications. In fact, the literature suggests that treatments with natural compounds inducing morphological modifications have to be continued for longer periods [28]. Our results show that none of the treatments affected plasma glucose and triglycerides, which in our study were not significantly different in WKYs and SHRs. In the literature, there are conflicting data on glucose and triglyceride plasma levels in these two strains [30,31].

It is well known that the onset and development of hypertension are closely related to inflammation [32,33]. Coherently, plasma TNF-α was significantly higher in SHRs than in WKYs, while SHRs treated with Gs had significantly lower plasma levels of TNF-α than SHRs treated with vehicle, and IL-1β and IL-6 were undetectable. Dietary supplementation with Gs and tomatine appeared to be safe, as no significant effects were recorded on weight gain, appearance of the animals, or the content and activity of liver microsomal CYP [34]. Tomatine could be toxic at high doses, as described in the literature, which is why a dose below the toxicity threshold was chosen for this study [35].

Several studies indicate that drug efficacy may be modified by meals consumed concomitantly, as food matrices and bioactive compounds may interfere at different phases of the pharmacokinetic process [36]. Food molecules may interfere with enzymes involved in drug-metabolising systems, which in turn cause variable and limited or increased drug bioavailability [37]. Interestingly, Tsujimoto et al. [38], showed that different vegetable juices, such as cabbage, onion and green pepper, promoted a time-dependent inhibition of CYP3A4 in vitro, while tomato juice did not affect this activity. Liu et al. 2009 [39] showed no differences in ETR-*O*-dealkylase and NADPH-quinone oxireductase activities in control rats and rats fed with a diet containing 10% tomato powder; the same results were demonstrated feeding SHRs with *Tenebrio molitor* defatted larvae diet [40]. Our results are in line with these studies, as Gs and tomatine did not affect the different CYP activities. This evidence suggests that tomato does not interfere with the pharmacokinetics of drugs, unlike other vegetables, which may modulate drug-metabolising systems.

The daily amount of tomato extract administered to the rats was quite high, corresponding roughly to 100 g of fresh tomatoes per day. This may make it feasible to recover waste products of the tomato processing industry [41] to prepare dietary supplements to combine daily with a healthy diet and lifestyle.

In conclusion, the results suggest that Gs obtained from the Camone tomato variety had beneficial effects on systolic blood pressure and correlated parameters in hypertensive rats. The antihypertensive effects of tomato are a cheap and easy way to restore normal blood pressure and to avoid or delay drug therapy in patients with mild hypertension [18]. The present results provide clear evidence that a diet supplemented with Gs delays the development of hypertension, limiting blood pressure increase, and that the tomato is a rich source of healthy bioactive components.

## 4. Materials and Methods

### 4.1. Reagents

Analytical grade reagents and standards for chemical characterisation and HPLC grade solvents were purchased from Sigma-Aldrich (Milan, Italy), unless otherwise stated. Solutions were prepared by dissolving the compounds in distilled deionised water obtained from an Elix 10^®^–MilliQ^®^ water purification system (Millipore, Bedford, MA, USA).

### 4.2. Tomato Samples and Chemical Characterisation

**Figure 6:**
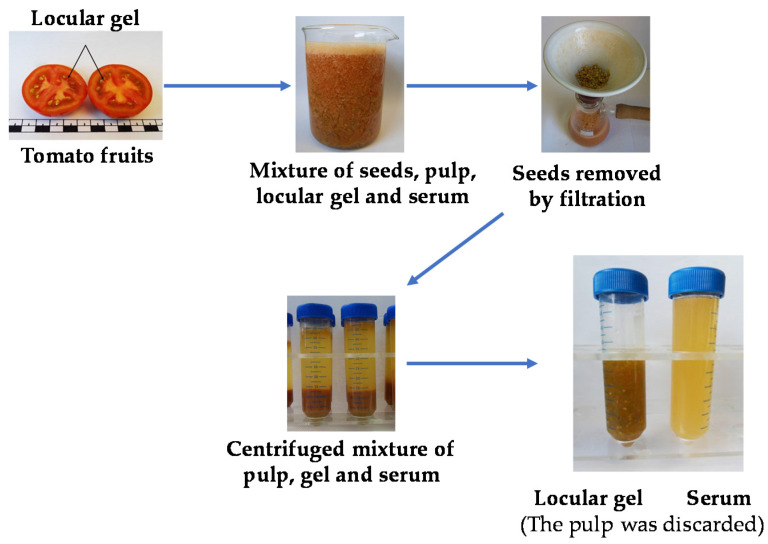
Scheme of locular gel and serum production from tomato fruits. Gs samples of Camone were extracted in triplicate by optimised solid-liquid ultrasound assisted protocols as previously reported [15]. The extracts were tested for total antioxidant capacity on the basis of quenching ability against the radical DPPH˙ and radical cation ABTS^+^, detected photometrically. Antioxidant capacity was expressed as Trolox Equivalent (TEAC) and the final results were expressed as μmol Trolox Equivalent (TE)/kg fresh weight (fw) for both TEAC/ABTS and TEAC/DPPH measurements. The methods were those reported in [42,43], with some modifications [15].

*Lycopersicum esculentum* L., Camone variety, was chosen on the basis of a previous chemical characterisation [15] and fruit size. Tomatoes were purchased from a local supplier, ensuring that the producing farm was always the same (Pachino, Sicily, Italy). They were obtained in May, when partly ripe. A stock of fruits was processed every week (for a total of 4 stocks in 4 weeks). After washing, the tomatoes were halved transversely and locular gel and the serum, were gently separated from the rest of tomato, paying attention to avoid seeds and pulp impurities, as previously described (see also Figure 6) [15]. The gel was then lyophilised to preserve its characteristic (5Pascal LIO-5P freeze-dryer; −51 ± 2 °C, 1.3 ± 0.3 mbar, 5 days), finely powdered in a mortar and stored in the dark at −20 ± 1 °C, until use. The serum (liquid) was collected in polyethylene containers and stored at −20 ± 1 °C. Subsequently, aliquots by 100 mg of lyophilised gel were reconstituted in a volume by 3 mL of serum for in vivo studies (oral administration of rats, gavage; 1:30 gel weight/serum volume (gel/serum, Gs).

Aliquots of serum were also lyophilised, stored at −20 ± 1°C, and used within 1 month for analytical characterisation.

To obtain the phenol profile, the extracts were tested for total polyphenol content by the photometric Folin–Ciocalteu assay [44] with some modifications [15] (using gallic acid as calibration reference, and expressing final results as mg Gallic Acid Equivalent (GAE)/kg fw). Selected polyphenols (hydroxycinnamic acids and flavonoids) were also quantitatively determined by HPLC-ESI-QqQ-MS/MS method: caffeic acid, chlorogenic acid, *p*-coumaric acid, rutin, kaempferol-3-O-rutinoside and a similar protocol was used to quantify tomatine content (α-tomatine and dehydrotomatine) in both lyophilised gel and serum extracts. Details on the extraction procedure and HPLC-ESI-QqQ-MS/MS methods are reported in a previous paper [15], and slight modifications were applied. Briefly, both lyophilised gel and serum aliquots were extracted by hydroalcoholic mixture (70% EtOH, 1% acidified by acetic acid) and the extraction was ultrasound assisted (15 min, 21 ± 2 °C; power, 120 W; sound frequency, 35 kHz; ultrasonic bath Sonorex Bandelin). Two subsequent extraction cycles were performed by 8 and 2 mL of mixture. After centrifugation, the supernatants were combined, and then dried under gentle nitrogen. Each sample was extracted in triplicates, and the dried extracts were stored at −20 ± 1 °C (dark) before subsequent analyses. The extracts were re-constituted in 80% MeOH solution, before the determination of selected polyphenols, α-tomatine and dehydrotomatine contents via HPLC-ESI-QqQ-MS/MS methods (HPLC Agilent 1200 Series, coupled with a mass spectrometer TSQ Quantum Access, equipped with electrospray ion source (ESI) and triple quadrupole (QqQ) analyser). The polyphenolic components were quantified by reverse phase liquid chromatography analysis (column: Phenomenex Kinetex biphenyl, 10.0 × 2.1 mm, core–shell, 5 μm particles, 100 Å pore, with a safe-guard pre-column, Phenomenex Phenyl, 4.0 × 2.0 mm; thermostated at 35 ± 1 °C) and linear gradient (A) H_2_O and (B) MeOH both acidified 0.1%(*v*/*v*) with formic acid, 500 μL/min elution flow rate [15], and injection volume by 20 μL. The ESI-MS conditions were optimised in negative ion current mode.

The method for the quantification of α-tomatine and dehydrotomatine was very similar, just a reverse phase column C18 (Phenomenex Luna C18, 5U, 250 × 4.6 mm, 5 μm particles, 100 Å pores) with safeguard pre-column (Phenomenex C18, 4.0 × 3.0 mm) was used for separation (thermostated at 30 ± 1 °C). The linear gradient was opportunely optimised at a 700 μL/min elution flow rate [15], and the injection volume was 20 μL. The ESI-MS conditions were optimised in positive ion current mode.

In both polyphenol and glycoalkaloid protocols, the quantification of selected species was carried out via SIM (single ion monitoring) and SRM (selected reaction monitoring) methods and via external calibration method, using genistein and tomatidine as internal standard, respectively. Calibrations showing correlation factors R^2^ > 0.980 were accepted for analyses, and LOD and LOQ values were those reported in Table 1. Each extract was injected in triplicates. More details and instrumental parameters are reported in Ref [15].

### 4.3. Animals

This study was approved by the Animal Ethics Committee of the University of Siena and the Italian Ministry for Health (n. 185/2015PR). Animal experimentation complied with European legislation on the use and care of laboratory animals (EU Directive 2010/63) and all efforts were made to minimise the number of animals used and their suffering. Nine-week-old spontaneously hypertensive male rats (SHRs) [45] and male Wistar Kyoto rats (WKYs) used as matched control, were purchased from Charles River (Charles River Italia S.p.A.). SHRs were used as they are considered suitable for studying the antihypertensive properties of drugs or dietary supplements as the development of hypertension is very similar to that in humans [24]. The age of rats was chosen considering that SHRs spontaneously develop hypertension about at X weeks of age. Animals were caged in groups of four and allowed to settle for a week under conditions of controlled temperature, 12 h light-dark cycle and food and water ad libitum. They were fed with a standard rat chow (53.5% carbohydrates, 18.5% protein, 3.0% fat; 6% crude fibres; 7% crude ash; 12% humidity; 4RF21 Mucedola, Milan, Italy). See also Table S1.

### 4.4. Experimental Design

At ten weeks of age, when hypertension starts spontaneously to develop, SHRs were randomly divided into four treatment groups (V, C, Gs, T) and treated daily by oral gavage (total administered volume 3 mL) for four weeks as detailed below:

- V: Vehicle (physiologic solution, *n* = 7);
- C: Captopril (50 mg/kg, *n* = 7), a well-known antihypertensive drug; dose chosen according to the literature [24];
- Gs: lyophilised gel reconstituted in serum (400 mg lyophilised gel in 12 mL serum, i.e., 12.4 g/kg, *n* = 8);
- T: Tomatine (8 mg/kg, *n* = 8).

Age-matched WKYs were divided into treatment groups (V, C, Gs, T) and treated in parallel as paired controls. The oral dose of tomatine was well below the reported toxic dose [46] for four-week treatment.

The animals were weighed twice a week; at the end of the four-week treatment period, urine was collected by housing the rats in a metabolic cage for 2 h in the morning. Overnight fasted animals were then anaesthetised by intraperitoneal injection of 15 mg/kg Zoletil® and 4 mg/kg Xylor® and blood was collected from the abdominal aorta. The rats were then killed by decapitation. At the end of the procedure, aorta, liver and heart were taken for further analysis. Rat hearts were placed in physiological solution to wash out residual blood, then freed of epicardial fat before being weighed by the same operator.

### 4.5. Physiological Parameters

A MultiCare® meter (Biochemical Systems International s.r.l., Arezzo, Italy) equipped with reagent strips was used for the measurement of blood glucose and triglyceride levels in serum, determined at the end of the experimental procedure and expressed in mg/dl. Urine was analysed using commercial test strips (Combi Screen 11 SYS Plus, Analyticon Biotechnologies AG, Germany). These strips include reagent pads showing a distinct color change in the clinically relevant range of bilirubin, urobilinogen, ketones, ascorbic acid, glucose, protein, haemoglobin, pH, nitrite, leukocytes and specific gravity.

### 4.6. Blood Pressure and Heart Rate

Systolic blood pressure (SBP) was measured before treatment (basal value) and twice a week by the non-invasive “tail-cuff” method [47], recorded with a digital PowerLab data acquisition system (PowerLab 8/30; ADInstruments, Castle Hill, Australia) and analysed using LabChart 7.3.7 Pro (Power Lab; ADInstruments). Three consecutive blood pressure readings were taken in the morning between 9am and 1pm after warming the body of the rat to 37 °C for 5 min. Heart rate (HR in beats per minute) was also recorded before the treatments (basal) and at the end of 4 weeks of treatment.

### 4.7. Histological Analysis

Immediately after removal, the thoracic aorta was cleaned, cut into rings and fixed in 10% buffered formalin for 3 days with a daily change of formalin solution. The samples were then dehydrated and embedded in paraffin wax. Serial sections, 8 µm thick, were cut with a microtome (Leica Microsystems, Milan, Italy), stained with Mayer’s hematoxylin and eosin and observed under a Nikon Eclipse E600 microscope (Nikon Instruments). Aorta thickness was measured blind by two expert operators using “Nis Elements v3AR” morphometric software (Nikon Instruments).

### 4.8. Inflammatory Cytokines

Blood samples were drawn into tubes containing gel for serum separation. Blood was allowed to clot for 30 min and centrifuged at 2000 g for 15 min within an hour of collection. Serum was then frozen at −20 °C until use. IL-6, IL-1β and TNF-α were measured in serum using ELISA kits (Millipore®) according to the manufacturer′s protocol.

### 4.9. Liver Cytochrome P450- and b5-content, NADPH-Cytochrome P450 Reductase Activity

Microsomal fractions were prepared from livers of overnight fasted rats, as previously reported [48]. Microsomes were washed and resuspended in KCl/ MOPS buffer (100 mM KCl, 20 mM NaCl, 1 mM MgCl_2_, 20 mM MOPS, pH 7.2) and kept in liquid nitrogen until use. The protein concentration in microsomal suspensions was determined by the method of Lowry [49] using BSA as standard.

CYP and cytochrome b5 contents were measured from the CO-difference spectra of the microsomal preparation at ΔA: 450–490 nm (ε: 91 mM) and ΔA:424–490 nm (ε: 112 mM), respectively [50]; NADPH-cytochrome P450 reductase activity was measured by following cytochrome c reduction at 550 nm according to Masters et al. [51] with a slight modification as previously reported [52].

### 4.10. Alkoxyresorufin Assay for Determination of Rat Liver Microsome CYP-Dependent-Activities

Pentoxyresorufin (PTR), ethoxyresorufin (ETR) and benzyloxyresorufin (BZR) are substrates dealkylated by different CYP isoforms to selective substrate markers: ETR for the CYP1A, PTR for the CYP 2B, and BZR for the CYP 1A, 2B, 2C and 3A families [53,54,55,56]. The incubation mixture (total volume 100 µl) contained the following components (final concentration): Phosphate–Buffer, pH 7.4 (100 mM), EDTA (0.1 mM), rat liver microsomes (1 mg protein/ml), NADPH (0.215 mM) and probe substrate ETR (10 µM), PTR (10 µM) or BZR (10 µM). Reactions were started by adding NADPH and after 45 min incubation at 37 °C, the amount of resorufin formed was measured with a Fluoroskan Ascent fluorimeter (Thermolabsystem, Helsinki, Finland) at an excitation wavelength of 544 nm and emission wavelength of 590 nm. The amount of fluorescence detected was expressed with reference to a standard curve made with pure resorufin (0.3–1 µM) as previously reported [57].

### 4.11. Statistical Analysis

Tomato analytical determinations were carried out in triplicate (*n* = 27). Results of chemical characterisation data are reported as mean ± SD and compared for significant differences by one-way ANOVA followed by Tukey′s post-test.

Animal results data are reported as mean ± SEM and compared for significant differences by Student t-test, by one-way or two-way ANOVA, as appropriate, followed by Bonferroni post-test (for separated groups of animal, i.e., WKYs, SHRs) (GraphPad Prism version 5.04, GraphPad Software Inc., San Diego, CA, USA).

The level of statistical significance (*p*) was set at 0.05 for most comparisons, if not differently specified.

## Figures and Tables

**Table 1:** Polyphenols and glycoalkaloids content in gel and serum from Camone tomatoes sampled in four different weeks.

	**Locular Gel**			
	**Week 1**	**Week 2**	**Week 3**	**Week 4**
**Caffeic acid**	5.13 ± 0.04 ^a^	9.85 ± 0.17 ^b^	6.12 ± 0.49 ^c^	8.11 ± 0.24 ^d^
**Chlorogenic acid**	68.2 ± 1.7 ^a^	90.7 ± 3.5 ^b^	53.4 ± 2.3 ^c^	29.5 ± 0.6 ^d^
***p*** **-Coumaric acid**	0.200 ± 0.001 ^a^	0.513 ± 0.007 ^b^	0.233 ± 0.004 ^c^	0.368 ± 0.009 ^d^
**Rutin**	0.717 ± 0.021 ^a^	0.321 ± 0.007 ^b^	0.210 ± 0.008 ^c^	trace
**α-Tomatine**	61.7 ± 0.9 ^a^	9.05 ± 0.37 ^b^	13.7 ± 0.3 ^c^	4.41 ± 0.32 ^d^
**Dehydrotomatine**	7.30 ± 0.25 ^a^	2.04 ± 0.09 ^b^	2.80 ± 0.19 ^c^	0.78 ± 0.09 ^d^
	**Serum**			
	**Week 1**	**Week 2**	**Week 3**	**Week 4**
**Caffeic acid**	2.31 ± 0.04 ^a^	6.36 ± 0.52 ^b^	3.94 ± 0.17 ^c^	3.82 ± 0.04 ^c^
**Chlorogenic acid**	83.6 ± 3.0 ^a^	92.3 ± 4.7 ^b^	51.5 ± 1.0 ^c^	26.1 ± 0.8 ^d^
***p*** **-Coumaric acid**	nd	0.140 ± 0.017 ^a^	trace	trace
**Rutin**	0.281 ± 0.016 ^a^	0.163 ± 0.005 ^b^	0.112 ± 0.001 ^c^	trace
**α-Tomatine**	12.5 ± 0.5 ^a^	1.51 ± 0.04 ^b^	1.90 ± 0.09 ^c^	0.65 ± 0.04 ^d^
**Dehydrotomatine**	1.86 ± 0.05 ^a^	0.305 ± 0.048 ^b^	0.362 ± 0.001 ^c^	0.118 ± 0.001 ^d^

Data are reported as mean ± SD (*n* = 27) and are expressed as mg/kg fw. Values with different letters in the same raw are significantly different (ANOVA with Tukey test, *p* < 0.05). nd, not detected: <limit of detection (LOD); trace: <limit of quantification (LOQ). Caffeic acid, *p*-Coumaric acid, Rutin and α-Tomatine: LOD//LOQ = 0.10//0.03 mg/L standard. Dehydrotomatine: LOD//LOQ = 0.30//0.10 mg/L standard. Chlorogenic acid: LOD/LOQ = 0.50//0.20 mg/L standard.

**Table 2:** Concentrations of chlorogenic acid, caffeic acid and α-tomatine in Gs administered in the four weeks of gavage.

	Week 1	Week 2	Week 3	Week 4
**Chlorogenic acid**	1276 ± 37 ^a^	1470 ± 58 ^b^	832 ± 15 ^c^	431 ± 10 ^d^
**Caffeic acid**	48.2 ± 0.5 ^a^	116 ± 6 ^b^	72 ± 3 ^c^	78 ± 1 ^c^
**α-Tomatine**	396 ± 7 ^a^	54 ± 2 ^b^	78 ± 2^c^	25 ± 1 ^d^

Data are reported as mean ± SD (*n* = 27) and are expressed as μg/kg body weight of rats. Values with different letters in the same raw are significantly different (ANOVA with Tukey test, *p* < 0.05).

**Table 3:** Effects of different treatments on body weight and heart weight to body weight ratio in SHRs and WKYs.

Strain	Diet	Body Weight (g)		Heart Weight/Body Weight (× 10^−3^)
		Time 0	Week 4	Week 4
**WKYs**	Vehicle	262.4 ± 3.8	303.4 ± 3.7	3.83 ± 0.03
	Captopril	267.6 ± 4.8	304.2 ± 4.4	3.87 ± 0.43
	Gel/serum	266.0 ± 4.2	305.5 ± 8.4	3.60 ± 0.27
	Tomatine	262.2 ± 4.4	301.7 ± 4.4	3.68 ± 0.08
**SHRs**	Vehicle	239.2 ± 4.3	282.2 ± 8.1	4.20 ± 0.13
	Captopril	254.4 ± 2.9	292.4 ± 5.3	3.88 ± 0.10
	Gel/serum	246.5 ± 4.6	289.2 ± 5.9	3.98 ± 0.15
	Tomatine	249.0 ± 7.7	290.0 ± 7.7	3.93 ± 0.14

Rats were treated for 4 weeks with: vehicle, captopril 50 mg/kg, tomatine 8 mg/kg or gel/serum 12.40 g/kg. Data are reported as mean ± SEM (*n* = 7–8). No significant differences were observed between groups of treatments as assessed by ANOVA.

**Table 4:** Effects of different treatments on blood glucose, triglyceride levels and urine volumes in SHRs and WKYs.

	**WKY V**	**WKY C**	**WKY Gs**	**WKY T**
Glucose (mg/dL)	86.80 ± 5.48	88.00 ± 6.31	83.17 ± 6.26	78.83 ± 10.17
Triglycerides (mg/dL)	129.16 ± 9.48	119.60 ± 4.53	126.83 ± 9.85	125.33 ± 3.18
Urine volume (mL)	8.00 ± 0.10	13.50 ± 3.58	10.00 ± 0.82	9.00 ± 0.82
	**SHR V**	**SHR C**	**SHR GS**	**SHR T**
Glucose (mg/dL)	75.80 ± 5.56	75.80 ± 5.67	73.67 ± 5.94	72.50 ± 2.75
Triglycerides (mg/dL)	115.80 ± 4.81	126.80 ± 11.43	110.83 ± 7.95	132.83 ± 6.16
Urine volume (mL)	10.00 ± 3.79	9.00 ± 0.10	11.00 ± 1.78	11.00 ± 1.47

Rats were treated for 4 weeks with: vehicle, captopril 50 mg/kg; tomatine 8 mg/kg and gel/serum 12.40 g/kg. Data are reported as mean ± SEM (*n* = 7–8) and were measured at the end of 4 weeks of treatment. No significant differences were observed between groups as assessed by ANOVA. V: vehicle; C: captopril; T: tomatine; Gs: gel/serum.

**Table 5:** Cytochrome content and cytochrome P450 reductase activity in liver microsomes obtained from treated WKYs and SHRs.

Strain	Treatment	Cytochrome P450	Cytochrome b5	Cytochrome P450-NADPH-Reductase
		nmol/mg	nmol/mg	nmol/min/mg
**WKYs**	Vehicle	0.80 ± 0.13	0.29 ± 0.07	19.01 ± 4.06
	Captopril	0.76 ± 0.10	0.27 ± 0.04	13.90 ± 3.67
	Gel/serum	0.60 ± 0.06	0.32 ± 0.03	13.24 ± 2.27
	Tomatine	0.69 ± 0.13	0.32 ± 0.04	17.75 ± 1.44
**SHRs**	Vehicle	0.50 ± 0.17	0.25 ± 0.07	30.17 ± 7.08
	Captopril	0.68 ± 0.19	0.28 ± 0.05	40.27 ± 18.30
	Gel/serum	0.46 ± 0.11	0.23 ± 0.03	33.13 ± 7.27
	Tomatine	0.68 ± 0.13	0.32 ± 0.04	37.70 ± 14.34

Rats were treated for 4 weeks with: vehicle, captopril 50 mg/kg; tomatine 8 mg/kg; gel/serum 12.40 g/kg. Data are reported as mean ± SEM and were measured at the end of 4 weeks of treatment. No significant differences were observed between groups of the same strain, as assessed by ANOVA.

**Table 6:** Cytochrome P450-dependent monooxygenase activities in liver microsomes from treated WKYs and SHRs.

		SUBSTRATE		
Strain	Treatment	ETR	PTR	BZR
		pmol/min/mg	pmol/min/mg	pmol/min/mg
**WKYs**	Vehicle	9.09 ± 2.39	2.40 ± 0.21	2.14 ± 0.46
	Captopril	7.68 ± 1.77	1.88 ± 0.25	2.58 ± 0.43
	Gel/serum	9.23 ± 1.61	2.27 ± 0.41	2.98 ± 0.74
	Tomatine	9.76 ± 1.46	2.70 ± 0.64	2.55 ± 0.22
**SHRs**	Vehicle	7.57 ± 1.87	2.64 ± 0.65	4.25 ± 0.26
	Captopril	7.27 ± 1.27	2.39 ± 0.28	4.83 ± 0.52
	Gel/serum	7.81 ± 0.84	2.93 ± 0.46	4.55 ± 0.46
	Tomatine	8.60 ± 0.53	2.45 ± 0.29	5.01 ± 1.08

PTR, ETR and BZR were used as marker substrates for CYP 1A, CYP 2B and CYP 1A, 2B, 2C, 3A activities, respectively. *O*-dealkylase activities were assayed by plotting the time-trend of resorufin formation detected at λ_ex_ 544 and λ_em_ 590 nm as reported in the Materials and Methods. Animals were treated for 4 weeks with vehicle, captopril 50 mg/kg, tomatine 8 mg/kg or gel/serum 12.40 g/kg. Data are reported as mean ± SEM (*n* = 5–7). No significant differences were detected between groups of the same strain, as assessed by ANOVA. PTR: pentoxyresorufin, ETR: ethoxyresorufin, BZR: benzyloxyresorufin.

## Supplementary Materials

The following are available online, Table S1: Diet composition and ingredients used in the present study.
