# Supplementary material for: Chemical Characterisation and Antihypertensive Effects of Locular Gel and Serum of Lycopersicum esculentum L. var. “Camone” Tomato in Spontaneously Hypertensive Rats
Source: Molecules. 2020 Aug 18;25(16):3758. doi: 10.3390/molecules25163758 (PMC7464676; doi:10.3390/molecules25163758)
Supplement: Supplementary file 1 [file molecules-25-03758-s001.pdf]

Table S1. Diet composition and ingredients used in the present study

Mucedola 4RF21 Standard diet	
**Composition**	
Humidity	12%
Protein	18,5%
Fat	3%
Carbohydrate	53,5%
Crude Fibers	6%
Crude ash	7%
Vitamin A	U.I. 14400
Vitamin D3	U.I. 1260
Fe	mg 180
Mn	mg 54
Zn	mg 67.5
Cu	mg 11.7
I	mg 0.90
